# Mycorrhizal Communities and Isotope Signatures in Two Partially Mycoheterotrophic Orchids

**DOI:** 10.3389/fpls.2021.618140

**Published:** 2021-02-09

**Authors:** Hans Jacquemyn, Rein Brys, Michael Waud, Alexandra Evans, Tomáš Figura, Marc-André Selosse

**Affiliations:** ^1^Department of Biology, Plant Conservation and Population Biology, Department of Biology, Katholieke Universiteit Leuven, Leuven, Belgium; ^2^Research Institute for Forest and Nature, Geraardsbergen, Belgium; ^3^Institut de Systématique, Evolution, Biodiversité (ISYEB), Muséum national d'Histoire naturelle, CNRS, Sorbonne Université, EPHE, Paris, France; ^4^Faculty of Biology, University of Gdańsk, Gdańsk, Poland

**Keywords:** mixotrophy, mycorrhiza, Orchidaceae, photosynthesis, stable isotopes, trophic modes

## Abstract

Partial mycoheterotrophy, the ability of plants to obtain carbon from fungi throughout their life cycle in combination with photosynthesis, appears to be more common within the Plant Kingdom than previously anticipated. Recent studies using stable isotope analyses have indicated that isotope signatures in partially mycoheterotrophic plants vary widely among species, but the relative contributions of family- or species-specific characteristics and the identity of the fungal symbionts to the observed differences remain unclear. Here, we investigated in detail mycorrhizal communities and isotopic signatures in four co-occurring terrestrial orchids (*Platanthera chlorantha, Epipactis helleborine, E. neglecta* and the mycoheterotrophic *Neottia nidus-avis*). All investigated species were mycorrhizal generalists (i.e., associated with a large number of fungi simultaneously), but mycorrhizal communities differed significantly between species. Mycorrhizal communities associating with the two *Epipactis* species consisted of a wide range of fungi belonging to different families, whereas *P. chlorantha* and *N. nidus-avis* associated mainly with Ceratobasidiaceae and Sebacinaceae species, respectively. Isotopic signatures differed significantly between both *Epipactis* species, with *E. helleborine* showing near autotrophic behavior and *E. neglecta* showing significant enrichment in both carbon and nitrogen. No significant differences in photosynthesis and stomatal conductance were observed between the two partially mycoheterotrophic orchids, despite significant differences in isotopic signatures. Our results demonstrate that partially mycoheterotrophic orchids of the genus *Epipactis* formed mycorrhizas with a wide diversity of fungi from different fungal families, but variation in mycorrhizal community composition was not related to isotope signatures and thus transfer of C and N to the plant. We conclude that the observed differences in isotope signatures between *E. helleborine* and *E. neglecta* cannot solely be explained by differences in mycorrhizal communities, but most likely reflect a combination of inherent physiological differences and differences in mycorrhizal communities.

## Introduction

Partial mycoheterotrophy (PMH), a form of mixotrophy that enables plants to obtain carbon from fungi (i.e., a net flow of C from a fungus to a plant) throughout their life cycle in combination with photosynthesis (Gebauer and Meyer, 2003), appears to be common within the Plant Kingdom (Selosse and Roy, 2009; Merckx, 2013; Jacquemyn and Merckx, 2019), but has only recently received increased attention (e.g., Těšitel et al., 2018). PMH has been considered to be an intermediate evolutionary stage from which full mycoheterotrophy has evolved (Julou et al., 2005; Selosse et al., 2017; Jacquemyn and Merckx, 2019). However, recent studies have indicated that the evolution from partial mycoheterotrophy to full mycoheterotrophy may be costly and involves specific adaptations (Roy et al., 2013; Gonneau et al., 2014) and a switch toward fungal partners that are able to provide more carbon and/or nitrogen to the plants (Ogura-Tsujita et al., 2012; Yagame et al., 2016; Jacquemyn and Merckx, 2019). As a result, PMH is considered to be evolutionary metastable (Těšitel et al., 2018) and displays an almost continuous gradient between autotrophy and full mycoheterotrophy (Gebauer et al., 2016; Jacquemyn et al., 2017; Schiebold et al., 2017; Jacquemyn and Merckx, 2019). However, what ecological factors determine the extent of dependency on fungal carbon and nitrogen and whether this is affected by the identity of the fungi involved in the mycorrhizal symbiosis, remains to some extent unclear (Hynson et al., 2013).

Previous research has shown that differences in stable isotope composition and N concentrations were related to plant family-specific physiological interactions with fungi and their environments (Hynson et al., 2016) as well as with environmental and developmental aspects in a given species (e.g., Matsuda et al., 2012; Roy et al., 2013; Gonneau et al., 2014). In orchids, light availability and fungal identity have been identified as important drivers of differences in carbon and nitrogen isotope abundance patterns. Partial mycoheterotrophy appears to be common in species associated with ectomycorrhizal fungi (e.g., Bidartondo et al., 2004; Selosse et al., 2004; Schiebold et al., 2017), and these species tend to be more flexible in responding to low-light conditions by increasing the proportional carbon gain from the fungal source than rhizoctonia-associated orchid species (Preiss et al., 2010; Liebel et al., 2015; Schweiger et al., 2019). Orchids associating with typical rhizoctonia fungi are usually not, or only weakly, enriched in heavy carbon and nitrogen isotopes (e.g., Stöckel et al., 2011; Jacquemyn et al., 2017; Schweiger et al., 2018). However, recent analyses using hydrogen stable isotope abundance have shown that rhizoctonia-associated orchids receive fungal organic matter as well (Gebauer et al., 2016; Schiebold et al., 2018; Schweiger et al., 2018, 2019), but precise characterization of the net flow remains to be established.

Further evidence for interactions with fungi affecting nitrogen and carbon uptake from fungi was given by Schiebold et al. (2017), who investigated carbon and nitrogen isotope abundance in fourteen *Epipactis* species. They showed that the type of mycorrhizal fungi found in the roots was related to variation in ^15^N enrichment of leaf tissue, suggesting that variation in mycorrhizal communities drives variation in isotope signatures. Because ectomycorrhizal fungi are generally involved in trophic interactions with surrounding trees, it was suggested that they provide larger and more stable supplies of fungal carbon and nitrogen than the more typical rhizoctonia fungi (Bruns et al., 2002; Selosse and Martos, 2014). However, recent research using high-throughput sequencing has shown that the mycorrhizal communities associating with orchid roots often consist of complex assemblages of multiple fungi from different fungal families and genera (Jacquemyn et al., 2015, 2016, 2017; Waud et al., 2017), all of which can potentially contribute to the resource budget of the plant. For example, adult plants of *Liparis loeselii* associated with various fungi of Thelephoraceae, Sebacinaceae, Russulaceae, Tulasnellaceae, Psathyrellaceae and Inocybaceae (Waud et al., 2017), thus combining rhizoctonia and ectomycorrhizal fungi. Roots of *Epipactis palustris* showed associations with a large number of fungal strains of different families, including Tulasnellaceae, Ceratobasidiaceae, Sebacinaceae and Thelephoraceae, and to a lesser extent Inocybaceae, Cortinariaceae, and Herpotrichiellaceae (Jacquemyn et al., 2016; Jacquemyn and Merckx, 2019). Similarly, *Epipactis helleborine* harbors ectomycorrhizal and rhizoctonia fungi (Jacquemyn et al., 2016; Xing et al., 2020), and in culture conditions the latter can be the sole associates (May et al., 2020). These results indicate that the mycorrhizal communities associating with orchids can be diverse and that a strict distinction in fungal syndromes cannot be easily made. They also question the previous finding that isotope signatures can be directly related to variation in mycorrhizal communities (Schiebold et al., 2017).

Here we investigated in detail mycorrhizal communities and isotope signatures in four co-occurring orchid species (*Platanthera chlorantha, Epipactis helleborine* and *E. neglecta* and the fully mycoheterotrophic *Neottia nidus-avis*). Photosynthetic rates and stomatal conductance were also measured in the partial mycoheterotrophic *Epipactis* species. Previous research has shown that *P. chlorantha* mainly associates with Ceratobasidiaceae (Bidartondo et al., 2004; Esposito et al., 2016), while *N. nidus-avis* associates mainly with *Sebacina* species (McKendrick et al., 2002; Selosse et al., 2002; Bidartondo et al., 2004, Těšitelová et al., 2015; Yagame et al., 2016). *Epipactis helleborine*, on the other hand, is a mycorrhizal generalist that associates with a wide diversity of fungi from different families, including Tuberaceae, Thelephoraceae, Russulaceae, Inocybaceae and Sebacinaceae as well as some rhizoctonias (Bidartondo et al., 2004; Bidartondo and Read, 2008; Ogura-Tsujita and Yukawa, 2008; Jacquemyn et al., 2016; Suetsugu et al., 2017; May et al., 2020; Xing et al., 2020). Schiebold et al. (2017) have suggested that *E. neglecta* forms orchid mycorrhizas exclusively with the ectomycorrhizal species *Tuber excavatum*, but detailed investigations of the mycorrhizal communities associating with *E. neglecta* are currently lacking.

## Materials and Methods

### Study Species and Sites

We studied isotopic signatures and mycorrhizal communities in four orchid species (*Platanthera chlorantha, Epipactis helleborine, E. neglecta* and *Neottia nidus-avis*) co-occurring at two sites in the Belgian Ardennes. The first site (50°06'04”N; 5°09'24”E) comprised a beech forest bordering a spruce forest and contained the four species growing in close proximity. *Platanthera chlorantha, E. neglecta*, and *N. nidus-avis* were restricted to the beech forest, whereas *E. helleborine* mainly occurred in the spruce forest. The second site (50°06'09”N; 5°10'35”E) consisted of a mixed deciduous forest with oak (*Quercus robur*) and European hornbeam (*Carpinus betulus*) as dominant tree species and a mixed pine forest consisting of Scots pine (*Pinus sylvestris*) and oak. At this site, both *N. nidus-avis* and *E. neglecta* were found in the deciduous forest, while *E. helleborine* occurred in the mixed pine forest.

### Sampling

Sampling took place in July 2018. To assess variation in orchid mycorrhizal communities among the different orchid species, 100 m^2^ plots were established at various places in the forest and within each plot young roots of five replicate individuals were collected for each species, making sure that no substantial damage was caused to the root systems. All sampled plants were in the flowering stage at the time of sampling. After roots had been carefully excavated from the soil, they were transported to the laboratory and immediately surface sterilized (30 s submergence in 1% sodium hypochlorite, followed by three 30 s rinse steps in sterile distilled water). Subsequently, DNA was extracted from 0.25 g of root fragments using the UltraClean Plant DNA Isolation Kit as described by the manufacturer (Mo Bio Laboratories Inc., Solana Beach, CA, USA).

At the same time of the root sampling, leaves were taken from the same five individual plants per orchid species for isotope analyses. In the case of *N. nidus-avis*, no leaves are available and flowering stalks were collected. Additionally, leaves were collected in the same 100 m^2^ plots from herbaceous autotrophic plants growing under the same light conditions and at the same distance from the soil as the orchids as a baseline for later analyses. Autotrophic reference plants included *Fragaria vesca, Helleborus foetidus, Vincetoxicum hirundinaria*, and *Rubus fruticosus* (site 1), and *Geum urbanum, Lamium galeobdolon, Mercurialis perennis, Rubus fruticosus, Stachys sylvatica* and the N-fixing *Vicia sepium* (site 2).

### Molecular Analyses

DNA samples were subjected to PCR amplification using sample-specific barcode-labeled versions of the primers ITS86F (5′-GTGAATCATCGAATCTTTGAA-3′) (Turenne et al., 1999) and ITS4 (5′-TCCTCCGCTTATTGATATGC-3′) (White et al., 1990), generating amplicons that cover the hypervariable ITS2 region (Waud et al., 2014). We used the dual-indexing strategy of Kozich et al. (2013) to amplify the samples. Two replicate PCR reactions were performed and combined for each sample in reaction volumes of 25 μl containing 1x Titanium Taq PCR buffer, 150 μM of each dNTP, 0.5 μM of each primer, 1 × Titanium Taq DNA polymerase (Clontech, Saint-Germain-en-Laye, France) and 2 μl 10-times diluted DNA. The reaction was initiated by denaturation at 94°C for 120 s, followed by 30 cycles of denaturation at 94°C for 45 s, annealing at 59°C for 45 s and elongation at 72°C for 45 s followed by a final elongation at 72°C for 10 min. Amplicons were then purified using Agencourt AMPure XP magnetic beads (Beckman Coulter Genomics GmbH, South Plainfield, UK) according to the manufacturer's instructions and duplicates were combined. After the purified products had been quantified using a Qubit High Sensitivity Fluorometer kit (Invitrogen, Carlsbad, CA, USA), amplicons from each sample were combined at equimolar concentrations into an amplicon library. Subsequently, the library was subjected to an ethanol precipitation and loaded on an agarose gel. The band of the expected size (c. 400 bp) was excised and the QIAquick Gel Extraction Kit (Qiagen, Hilden, Germany) was used to purify the DNA again. Lastly, the DNA concentration was measured again and the library was diluted to 2 nM and sequenced at the Center for Medical Genetics (University of Antwerp, Antwerp, Belgium) using an Illumina MiSeq sequencer with v2 500 cycle reagent kit (Illumina, San Diego, CA, USA).

Sequences were received for each sample as de-multiplexed FASTQ files representing forward and reverse paired-end reads. Paired-end reads were merged and combined reads with a total expected error threshold above 0.5 were discarded using USEARCH (v10.0.240) (Edgar, 2013). The “classify.seqs” and “remove.lineage” commands in mothur (v. 1.36.1) were used to identify and remove potential mitochondrial, chloroplast, archaeal and eukaryote contaminants (Schloss et al., 2009). Remaining sequences were grouped into operational taxonomic units (OTUs) based on a 3% sequence dissimilarity cut-off using the UPARSE greedy algorithm in USEARCH, during which chimeric sequences and global singletons (i.e., OTUs with only one sequence represented in the entire data set) were removed (Edgar, 2013). Subsequently, the taxonomic origin of each OTU was determined with the “sintax” algorithm implemented in USEARCH (Edgar, 2016) in conjunction with the UNITE fungal database version 7.2 (22.08.2018) trained for the fungal ITS-2 region (Kõljalg et al., 2013). Taxonomic assignments were considered reliable when bootstrap confidence values exceeded 0.80.

### Stable Isotope Analyses

Samples were ground in 2 mL Eppendorf tubes in a ball mill (MM200, Retsch Gmbh, Haan, Germany) and analyzed for ^13^C/^12^C and ^15^N/^14^N ratios using a Thermo Flash 2000 elemental analyser coupled to a ThermoFinnigan DeltaV Advantage Continuous-Flow Isotope-ratio mass spectrometer. Relative abundances of the stable isotopes (δ values) were calculated as follows: δ^13^C or δ^15^N = (*R*_sample_ /*R*_standard_ − 1) × 1,000 (%0), where *R*_sample_ is the ^13^C/^12^C or ^15^N/^14^N ratio of the sample, and *R*_standard_ is the ^13^C/^12^C ratio of Vienna Pee Dee Belemnite or ^15^N/^14^N ratio of atmospheric N_2_, respectively. As internal standard we used alanine (δ^13^C = −22.16 ± 0.05%0; δ^15^N = 0.59 ± 0.05%0) and the average and standard deviation of the replicated measurements of this standard were −26.65 ± 0.05%0 for ^13^C and 0.81 ± 0.08%0 for ^15^N. As primary standards, we used caffeine IAEA-600 (δ^13^C = −27.77 ± 0.04%0) and ammonium sulfate IAEA-N-1 (δ^15^N = 0.40 ± 0.20%0). To compare isotope signatures between sites, normalized enrichment factors were calculated as ε = δ_S_ – δ_REF_, where δ_S_ is a single δ^13^C or δ^15^N value of an orchid species or an autotrophic reference plant, and δ_REF_ is the mean value of all autotrophic reference plants occurring at the site (Preiss and Gebauer, 2008). Following Hynson et al. (2013), the percentage of mycoheterotrophic C gain in biomass was calculated for both partially mycoheterotrophic orchids as *x* = [(δC_P_ – δC_AT_) / (δC_NNA_ – δC_AT_)] × 100 %, where δC_P_, δC_NNA_, and δC_AT_ are the mean δ^13^C values of the focal plant, *N. nidus-avis* (reference for mycoheterotrophic biomass), and surrounding autotrophic reference plants, respectively.

### Photosynthetic Rates and Stomatal Conductance

Prior to the sampling of the roots and leaves, a Licor LI-6800 Portable Photosynthesis System (PPS) was used to assess photosynthetic rates and stomatal conductance in the two partial mycoheterotrophic orchids. For five plants of each species, we monitored one leaf using the PPS for 30 min and determined the natural variability in stomatal conductance and CO_2_ assimilation. Photosynthesis stabilization generally took place after 15 min. All measurements were done on bright, sunny days between 10:00 and 12:00 in July 2019, on the same day in each population.

### Data Analysis

Based on read abundance of all detected fungi, variation in fungal community composition among sampled individuals of the different orchid species was visualized using non-metric multidimensional scaling (NMDS) in the R software package vegan (Oksanen et al., 2013) with the Bray-Curtis coefficient as distance measure. A Venn diagram using the R package VennDiagram was created to assess the overlap in mycorrhizal OTUs between the different orchid species. To test the hypothesis that mycorrhizal communities differed between species and sites, we performed permutational analysis of variance (PERMANOVA; Anderson, 2001) using the “adonis” function in the software package vegan (Oksanen et al., 2013). Both species and site were included as fixed factors in the analysis. Finally, species indicator analysis was used to investigate whether ectomycorrhizal fungi that were significantly associated with one of the investigated orchid species could be identified. The “multipatt” function in the R package indicspecies (De Cáceres et al., 2010) was used to define fungal OTUs that associate with a particular orchid species.

Normalized enrichment factors for ^13^C were plotted against those for ^15^N for each sampled orchid species and the autotrophic reference plants. For orchid species sampled at two different sites, a Student's *t*-test was used to test whether the mean δ^13^C or δ^15^N differed between sites. Analysis of variance (ANOVA) or its non-parametric alternative (Kruskal-Wallis test) was used to evaluate differences in mean δ^13^C and δ^15^N among species from a given site. If the null hypothesis (no difference between means) was rejected, Tukey's honestly significant difference (HSD) test was used to make pairwise multiple comparisons of the means. The alpha type I error threshold was set at 0.05. A Student's *t*-test was also used to see whether stomatal conductance and photosynthesis differed between the two partial mycoheterotrophic orchid species. All statistical analyses were performed using the R environment for statistical computing (R Development Core Team, 2013).

## Results

### Mycorrhizal Communities

In total, Illumina Miseq sequencing generated 2,069,182 sequences (2053 OTUs). After quality filtering, removal of unidentifiable OTUs, and rarefaction to 10,000 sequence per sample, the final data set comprised 1,706 fungal OTUs (345964 sequences), of which 70 (191026 sequences – 55.2%) were assigned to putatively orchid mycorrhizal OTUs according to Dearnaley et al. (2012) and information from previous studies that detected mycorrhizal fungi from the roots and protocorms of these and related orchid species (Supplementary Table 1). These belonged to various fungal genera, including *Cenococcum* (2 OTUs), *Ceratobasidium* (4 OTUs), *Cortinarius* (12 OTUs), *Exophiala* (3 OTUs), *Hebeloma* (1 OTU), *Inocybe* (6 OTUs), *Lactarius* (2 OTUs), *Leptodontidium* (1 OTU), *Mycena* (1 OTU), *Peziza* (1 OTU), *Russula* (6 OTUs), *Sebacina* (12 OTUs), Suillus (1 OTU) *Tomentella* (15 OTUs), *Tricholoma* (1 OTU), and *Tuber* (2 OTUs). Representative sequences for each mycorrhizal OTU found in this study were submitted to GenBank under the Accession Numbers MW364287 – MW364356.

**Figure 1:**
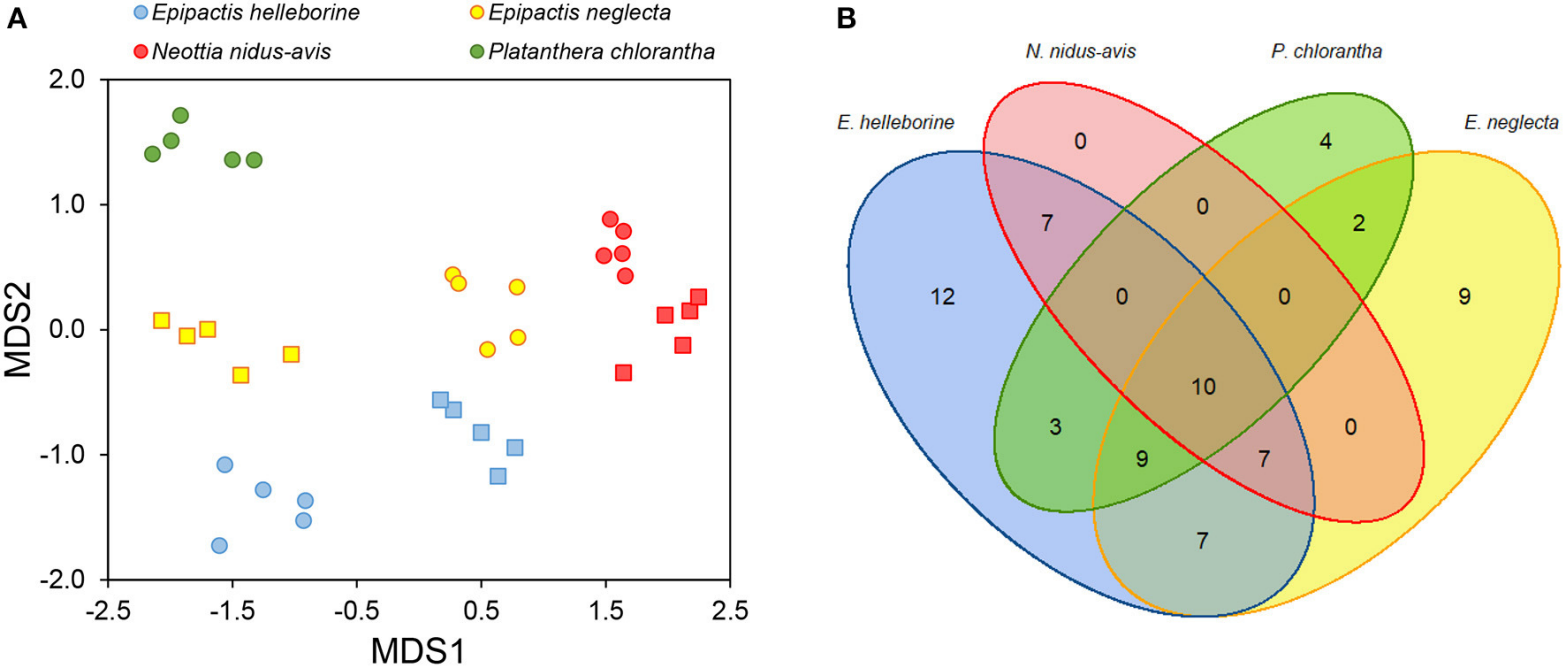
Variation in mycorrhizal communities associating with four different orchid species (*Epipactis helleborine, E. neglecta, Neottia nidus-avis* and *Platanthera chlorantha*). **(A)** NMDS graph displaying variation in fungal community composition between individuals of *E. helleborine* (blue), *E. neglecta* (yellow), *P. chlorantha* (green) and *N. nidus-avis* (red) sampled at two sites (site 1: dots, site 2: squares). **(B)** Venn diagram showing overlap in mycorrhizal OTUs between the sampled orchid species.

All individuals of the investigated orchid species associated with a large number of fungal OTUs [average number ± SD of OTUs detected per individual plant: 16.8 ± 1.6 (*P. chlorantha*); 24.1 ± 4.3 (*E. helleborine*); 21.6 ± 4.7 (*E. neglecta*); 9.0 ± 1.5 (*N. nidus-avis*)]. When only considering dominant OTUs (i.e., OTUs that represent more than 5% of the total number of reads), between 2.2 ± 0.8 (*N. nidus-avis*) and 4.6 ± 1.0 OTUs (*E. helleborine*) were observed per individual plant. Mycorrhizal community composition differed significantly among orchid species (pseudo-*F* = 11.18, *p* < 0.001) (Figure 1A), despite the fact there was considerable overlap in OTUs between species (Figure 1B). Eleven OTUs were found in all four species, but they all occurred with a low number of sequences. These belonged to the genera *Exophiala, Sebacina, Cenococcum, Ceratobasidium, Tuber, Cortinarius*, and *Inocybe*. For species that were sampled at the two sites, there was also a significant site effect (pseudo-*F* = 13.22, *p* < 0.001), but the magnitude of the site effect depended on the species (pseudo-*F* = 15.50, *p* < 0.001).

**Figure 2:**
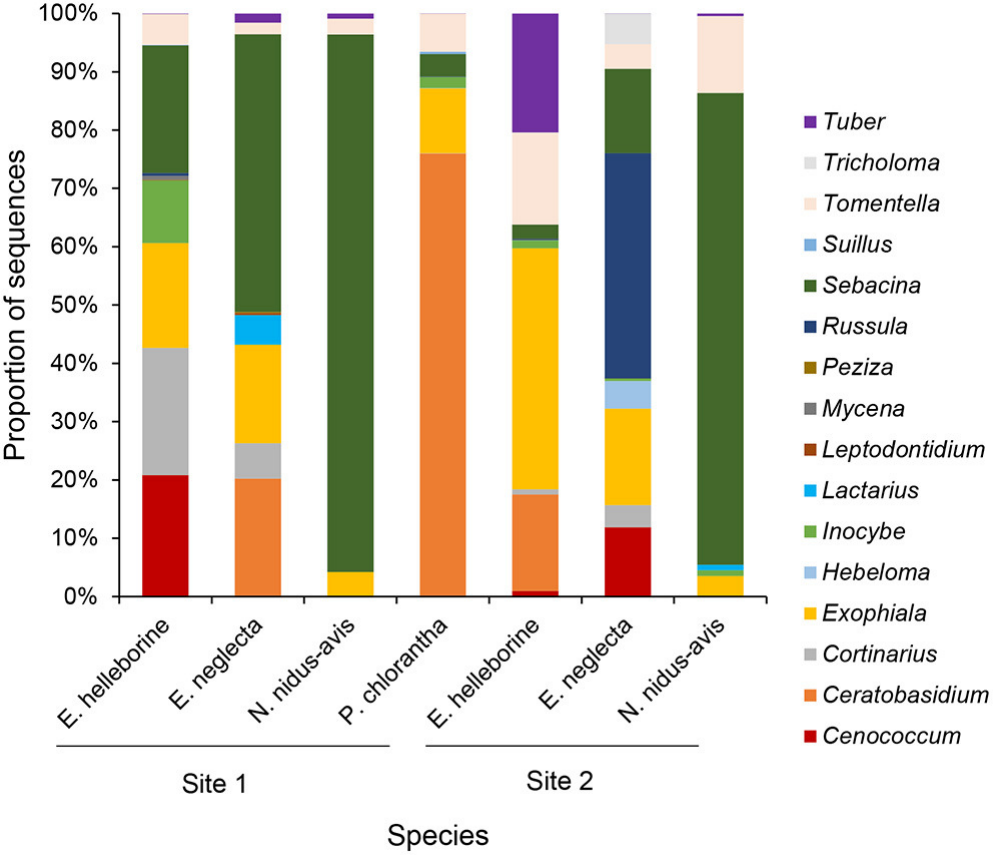
Bar charts representing the cumulative proportions of sequences belonging to different fungal genera observed in the sampled populations of each orchid species.

Species Indicator Analysis indicated that eighteen OTUs were significantly (*P* < 0.05) associated with *E. helleborine*, seventeen OTUs with *E. neglecta*, five with *N. nidus-avis* and eight with *P. chlorantha*. Mycorrhizal communities associating with *P. chlorantha* were dominated (75.8% of reads) by members of the genus *Ceratobasidium*, while those of *N. nidus-avis* were dominated (88.3% of reads) by members of the genus *Sebacina* (Figure 2). Additional fungi that were sporadically observed belonged to various genera of ectomycorrhizal fungi. Both *E. neglecta* and *E. helleborine* had more diversified fungal communities and no single fungal genus dominated their fungal community composition (Figure 2).

### Isotope Signatures

**Figure 3:**
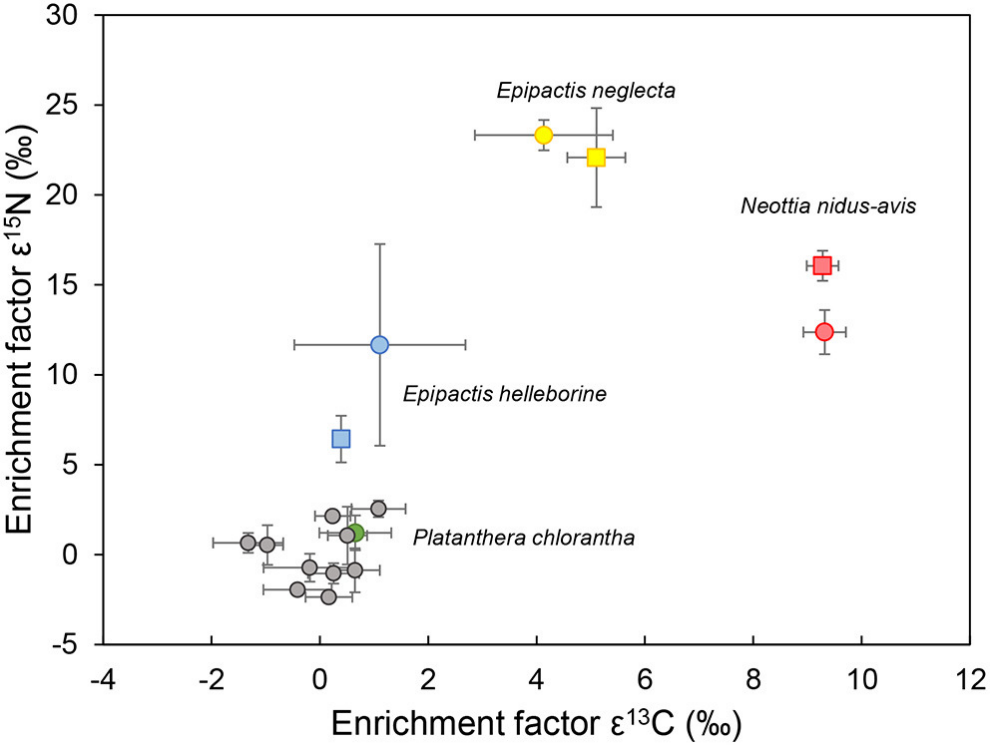
Enrichment factors ε^13^C and ε^15^N of four orchid species (blue: *Epipactis helleborine*, yellow: *E. neglecta*, red: *Neottia nidus-avis* and green: *Platanthera bifolia*) sampled at two different locations (site 1: dots; site 2: squares). Gray dots represent enrichment factors of autotrophic plants sampled at both locations. Values represent the mean of five individuals per species population, while bars represent standard deviations.

Isotope signatures clearly differed between the sampled species (Figure 3). The non-photosynthetic *N. nidus-avis* was significantly enriched in both ^13^C and ^15^N, whereas *P. chlorantha* did not show any signs of enrichment in ^13^C and ^15^N. *Epipactis neglecta* was significantly enriched in ^15^N at both sites and at one site in ^13^C, while *Epipactis helleborine* was significantly enriched in ^15^N, but not in ^13^C (Supplementary Figures 1, 2. The linear two-source mixing model approach (Hynson et al., 2013) indicated that *E. neglecta* showed 49.5 and 55.0% heterotrophy at sites 1 and 2, respectively, while this was only 4.2 and 13.2% for *E. helleborine*. These values only take into account the organic matter derived from ectomycorrhizal fungi, since gain from any rhizoctonia partner is undetectable on the basis of ^15^N or ^13^C enrichment (see Introduction).

### Photosynthetic Rates and Stomatal Conductance

**Figure 4:**
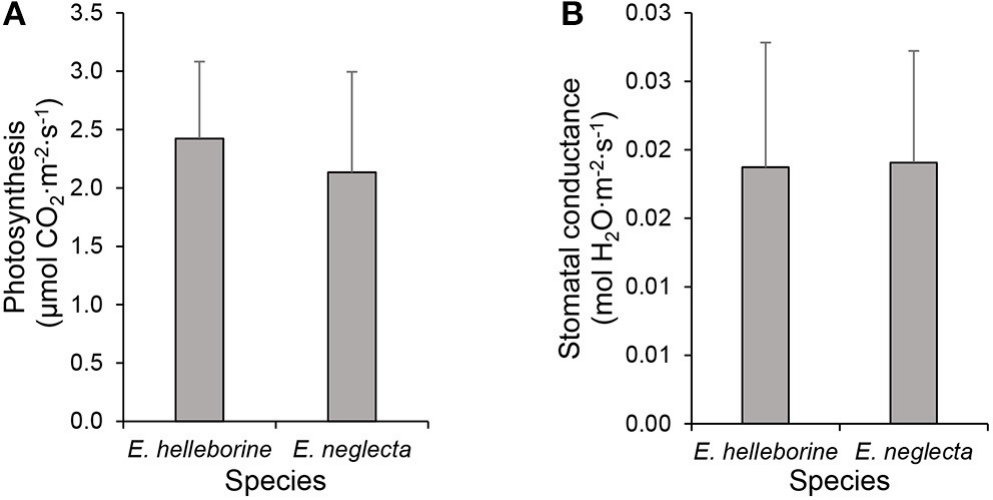
Instantaneous measurements of leaf gas exchanges on leaves of *Epipactis helleborine* and *E. neglecta* under natural growth conditions: **(A)** net CO_2_ assimilation and **(B)** water vapor stomatal conductance. Values represent the mean of five individuals per species population, while bars represent standard deviations.

Instantaneous measurements of leaf CO_2_ exchanges revealed that photosynthetic rates were low (mean ± SD: 2.42 ± 0.66 and 2.13 ± 0.86 μmol CO_2_·m^−2^·s^−1^ for *E. helleborine* and *E. neglecta*, respectively) and did not differ significantly (*t* = 0.60, *p* > 0.05) between individuals of both species (Figure 4A). Similarly, water vapor stomatal conductance values did not differ significantly (*t* = −0.06, *p* > 0.05) between the two species (Figure 4B).

## Discussion

### Mycorrhizal Communities

The investigated orchid species associated with a wide diversity of fungal OTUs. Results for *N. nidus-avis* and *P. chlorantha* were largely consistent with previous reports that have shown that both species mainly form mycorrhizas with *Sebacina* and *Ceratobasidium*, respectively (McKendrick et al., 2002; Selosse et al., 2002; Bidartondo et al., 2004; Těšitelová et al., 2015; Esposito et al., 2016; Yagame et al., 2016). Apart from these dominant fungi, several other fungi belonging to various mycorrhizal groups (*Exophiala, Inocybe* and *Tomentella*) were also sporadically observed in these species, yet it remains to be seen whether these additional fungi are truly mycorrhizal (i.e., form intracellular pelotons in the orchid roots) or simply represent endophytic fungi. This loose and non-mycorrhizal type of fungal colonization has recently been reported for some ectomycorrhizal fungi in non-ectomycorrhizal hosts (Schneider-Maunoury et al., 2020), including Tuberaceae, Thelephoraceae and Inocybaceae that were found here in *N. nidus-avis* and *P. chlorantha*. Overall, these findings seem to conform to a form of association that has recently been called “apparent generalism,” in which the orchid species appears to be a generalist because it associates with a large number of fungal species, but it is in fact a specialist of a few key, dominant fungi (Shefferson et al., 2019). Apparent generalism would suggest that once some minimum number of key fungal species is reached, extra fungal species convey no or few additional benefits to the orchid.

The situation appears to be different in the two *Epipactis* species, where no dominant fungus was observed, and associations with a large number of fungi were found. Unlike previous claims that *E. neglecta* forms orchid mycorrhizas exclusively with *Tuber* sp. (Schiebold et al., 2017), our results showed that this species, similar to other species in this genus (Bidartondo et al., 2004; Bidartondo and Read, 2008; Jacquemyn et al., 2016, 2017; Suetsugu et al., 2017), associates with a wide range of fungi, including members of the genera *Sebacina, Exophiala*, and *Cortinarius*. *Tuber* was observed but not exclusively in *E. neglecta*, and the highest number of sequences was observed in the roots of *E. helleborine*. Besides, significant differences in mycorrhizal communities were observed between the two sites that were sampled. This pattern of interaction specificity was recently coined “true generalism,” in which an orchid species associates with multiple hosts that overlap functionally and are geographically interchangeable based on opportunity for encounter, which leads to frequent host switching (Shefferson et al., 2019). In this case, it is generally assumed that the orchid opportunistically interacts with the fungi that are locally available. Further evidence for this mode of association was given in Xing et al. (2020), who investigated mycorrhizal associations in *E. helleborine* populations in Europe and Asia, spanning a geographic distance of >7,000 km. Consistent with a pattern of true generalism, rapid decline in community similarity with increasing distance and strong spatial turnover in mycorrhizal partners were observed, indicating frequent host switching across large geographic distances.

### Isotope Signatures

Isotope signatures of the investigated orchid species generally confirmed findings of previous studies (Bidartondo et al., 2004; Gonneau et al., 2014; Hynson et al., 2016; Schiebold et al., 2017; Suetsugu et al., 2017). *Platanthera chlorantha* was not significantly enriched in heavy carbon and nitrogen isotopes, while *N. nidus-avis* was significantly enriched in both isotopes. The two *Epipactis* species showed intermediate patterns of isotope enrichment. Although these results suggest that *P. chlorantha* is autotrophic, recent studies have indicated that orchids associated with typical rhizoctonia fungi of the families Ceratobasidiaceae, Serendipitaceae and Tulasnellaceae show hidden partial mycoheterotrophy, because these saprotrophic and endophytic fungi have relative abundances of ^15^N and ^13^C close to that of autotrophic plants (Gleixner et al., 1993; Selosse and Martos, 2014; Gebauer et al., 2016). Analyses of ^2^H and ^18^O content have shown that some rhizoctonia-associated orchids receive organic matter from their associated fungi (Gebauer et al., 2016; Schiebold et al., 2018; Schweiger et al., 2018), suggesting that rhizoctonia-mycorrhizal orchid species should be considered as partially mycoheterotrophic as well. However, characterization of the net flow has yet to be established and, up until now, no compelling evidence has been provided for the occurrence of achlorophyllous mutants or for individuals capable of living under the compensation point. Besides, it should be noted that so-called rhizoctonia-mycorrhizal orchids do not exclusively associate with rhizoctonia fungi, but often show associations with ectomycorrhizal fungi as well (Jacquemyn et al., 2017; Suetsugu et al., 2019), and even share some of the fungi associated with fully mycoheterotrophic orchids (Figures 1, 2).

*Epipactis helleborine* showed a near-autotrophic behavior, while *E. neglecta* was more mycoheterotrophic, particularly at site 2 where it was significantly enriched in both ^13^C and ^15^N. The linear two-source mixing model approach (Hynson et al., 2013) indicated that *E. neglecta* showed 49.5 and 55.0% heterotrophy at sites 1 and 2, respectively. The finding of near autotrophy in *E. helleborine* confirms recent results from pot cultures obtained by May et al. (2020). In this case, potted plants did not show higher N contents and higher isotopic (^13^C and ^15^N) abundances that are usually observed in partially mycoheterotrophic orchids. In contrast to what was observed in our study, the proportion of ectomycorrhizal fungi was low and a high percentage of rhizoctonias was found. Altogether, these findings suggest that the level of mycoheterotrophy in *E. helleborine* is variable, and to some extent depends on the prevailing environmental conditions.

The situation was different for *E. neglecta*. Consistent with previous research (Schiebold et al., 2017), this species was significantly enriched in ^15^N at both sites and in ^13^C at one site. Previous research has suggested that the high levels of ^15^N are due to the presence of *Tuber* or other ascomycetes (Schiebold et al., 2017). However, we did not find a higher prevalence of *Tuber* or ascomycetes in *E. neglecta*, suggesting that differences in mycorrhizal communities alone cannot explain the observed variation in isotope signatures in the two studied *Epipactis* species. Indeed, the mycorrhizal communities associated with *E. helleborine* and *E. neglecta* were more affected by site conditions than by the species themselves, whereas the isotope signatures were less affected by site conditions. This suggests that trophic modes in *Epipactis* are not necessarily driven by the mycorrhiza with which they associate, but may also reflect inherent differences in physiological traits of the species. Previous research has shown that developmental stage had a pronounced impact on the heterotrophy level in *Epipactis* (Roy et al., 2013; Gonneau et al., 2014), suggesting that physiological differences associated with the different developmental stages are more important than differences in mycorrhizal communities in determining trophic levels in *Epipactis*. Suetsugu et al. (2017) showed that achlorophyllous variants of *E. helleborine* var. *sayekiana* harbored similar mycobionts, mainly *Wilcoxina*, as green plants, but that the abundances of mycobionts were lower in the albino plants. Similar results have been found in other partially mycoheterotrophic orchids displaying albino plants such as *Cephalanthera damasonium* (Julou et al., 2005) and *E. microphylla* (Selosse et al., 2004). Nonetheless, albino plants were significantly more enriched in ^13^C and ^15^N than green plants due to the absence of photosynthesis.

In addition, gene expression analyses have shown that genes related to mycorrhizal symbiosis were upregulated in the albino variants (Suetsugu et al., 2017), together with genes involved in autolysis and amino-acid catabolism (Lallemand et al., 2019b). It is therefore not unlikely that differences in gene expression of genes related to carbon and nitrogen transport and use differ between *Epipactis* species and that these differences, rather than differences in mycorrhizal communities, explain the observed variation in isotopic signatures. Given that *E. helleborine* is a very widespread species that occurs in many different habitats and associates with a wide variety of fungi (Xing et al., 2020), selection on these genes is probably limited. Recent research has indeed shown that the plastid genome of *E. helleborine* retains a full set of photosynthetic genes without any evidence of selective relaxation (Lallemand et al., 2019a) and has intact photosynthetic abilities. In contrast, *E. neglecta* usually occurs in the understory of beech and oak forests, where very little light penetrates to the forest soil. Under these conditions, selection toward more efficient nutrient transport may be selected for, leading to more efficient carbon and nitrogen uptake from fungi.

## Conclusion

Establishing a firm relationship between mycorrhizal partners and isotope signatures appeared to be more difficult than previously suggested (Schiebold et al., 2017). Although the dominant partners varied, all investigated orchid species associated with a wide variety of fungi, supporting previous calls for carefully reporting minor traces of fungi that may be meaningful (Selosse et al., 2010, 2018). Autotrophic and fully mycoheterotrophic species had more specialized fungal communities than partially mycoheterotrophic species, which associated with a wide range of fungal partners, none of which was dominant. In partially mycoheterotrophic species, isotope signatures differed between the two *Epipactis* species, with *E. neglecta* being more mycoheterotrophic than *E. helleborine*, but differences in isotope signatures could not be related to differences in mycorrhizal communities, suggesting that inherent physiological differences contribute to the observed variation. In order to fully show the effect of mycorrhizal communities on isotope signatures without the confounding effects of possible physiological differences between species, experiments should be ideally conducted with a single orchid species that shows large variation in mycorrhizal communities. In this context, *E. helleborine* could be a perfect study system, as previous research has shown that its mycorrhizal communities differ substantially between European and Asian populations, the latter being dominated by ectomycorrhizal ascomycetes (*Tuber*), while the former are dominated by basiodiomycetous ectomycorrhiza (Xing et al., 2020).

## Data Availability Statement

The datasets presented in this study can be found in online repositories. The names of the repository/repositories and accession number(s) can be found in the article/Supplementary Material.

## Author Contributions

HJ and RB conceived the study. HJ, AE, and MW collected the data. MW performed the molecular analyses. M-AS and TF were in charge of the stable isotope analyses. HJ wrote the first draft. All authors contributed to the article and approved the submitted version.

## Conflict of Interest

The authors declare that the research was conducted in the absence of any commercial or financial relationships that could be construed as a potential conflict of interest.
