# Supplementary material for: Mycorrhizal Communities and Isotope Signatures in Two Partially Mycoheterotrophic Orchids
Source: Front Plant Sci. 2021 Feb 9;12:618140. doi: 10.3389/fpls.2021.618140 (PMC7901878; doi:10.3389/fpls.2021.618140)
Supplement: Supplementary file 1 [file Data_Sheet_1.PDF]

Appendix S1 Boxplots illustrating differences in $\delta^{13}C$ values between autotrophic reference plant species and *Plantanthera chlorantha*, *Epipactis helleborine, E. neglecta* and *Neottia nidus-avis* measured at two different sites. Different letters indicate significant differences according to analysis of variance (ANOVA) followed by Tukey C pairwise test ($P < 0.05$).

a)

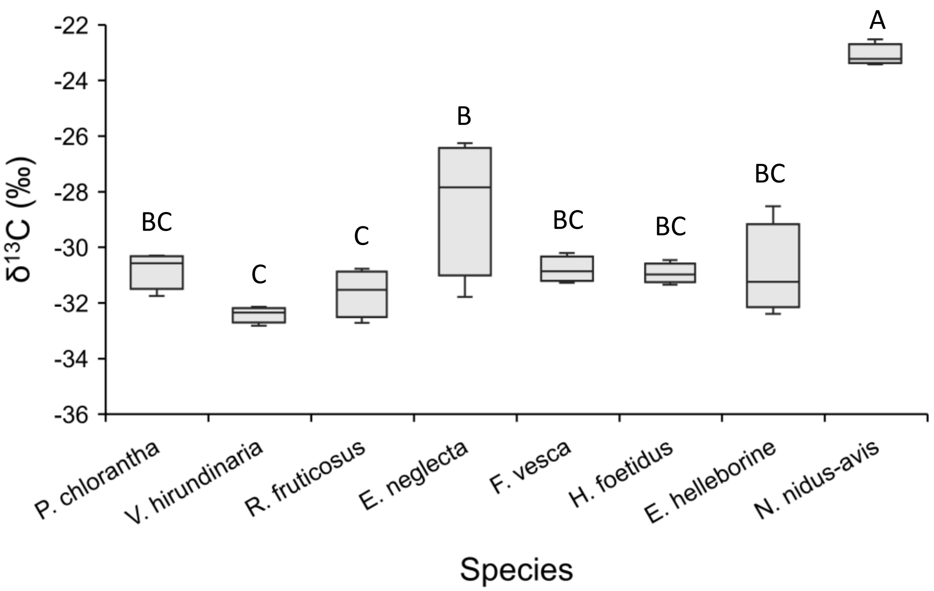

b)

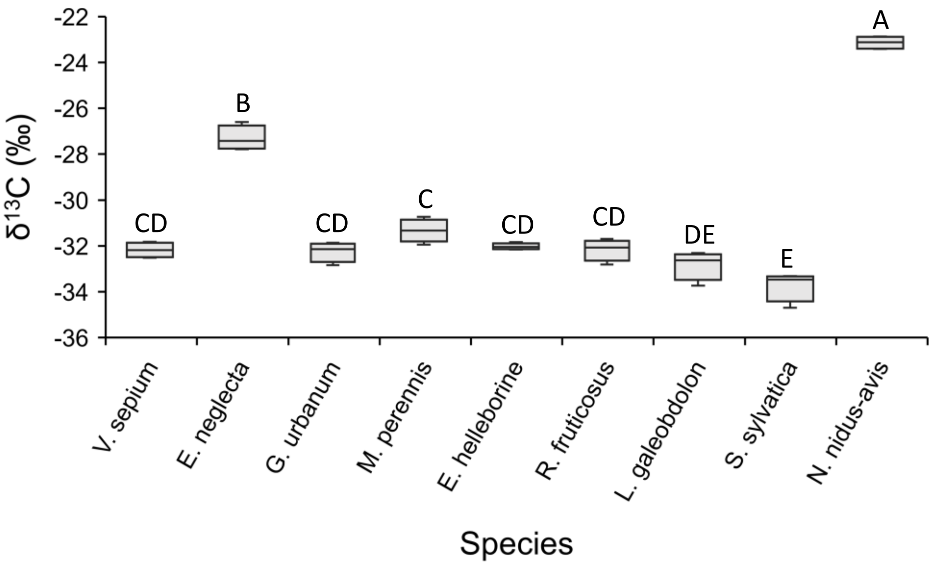

Appendix S2 Boxplots illustrating differences in δ^{15}N values between autotrophic reference plant species and *Plantanthera chlorantha*, *Epipactis helleborine*, *E. neglecta* and *Neottia nidus-avis* measured at two different sites. Different letters indicate significant differences according to a Kruskal-Wallis test followed by pairwise Wilcoxon tests ($P < 0.05$).

a)

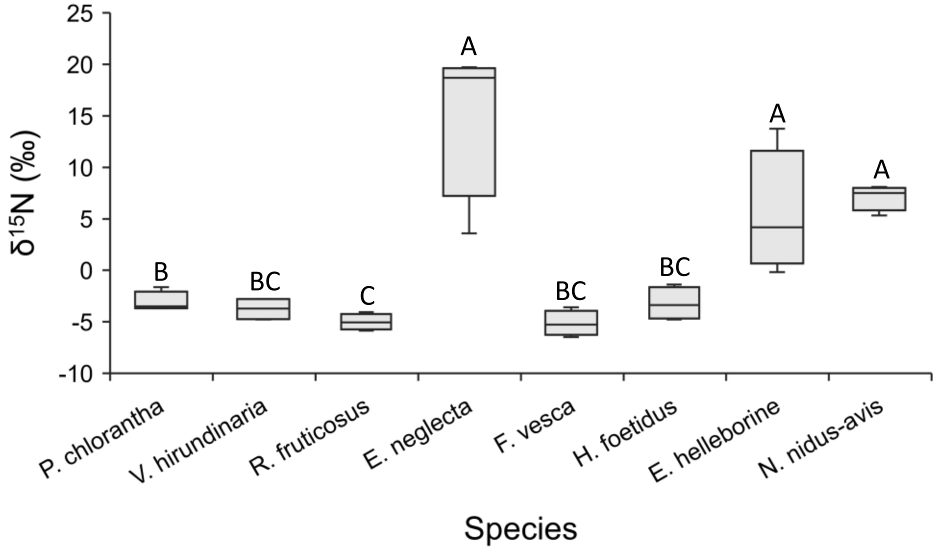

b)

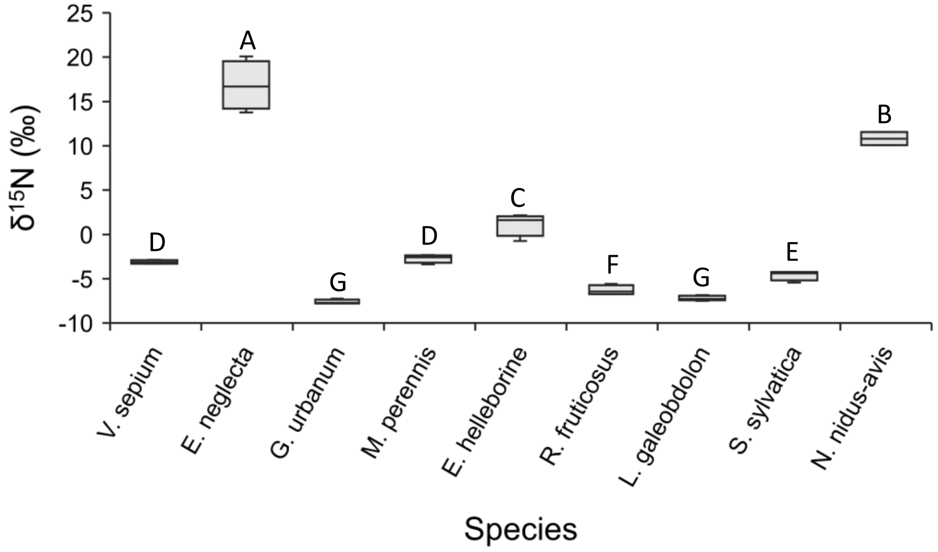

Appendix S3 δ^{13}C and δ^{15}N values of four orchid species and autotrophic reference plant species collected at two different sites.

**Species**	**δ^{13}C**	**δ^{15}N**
Site 1		
Epipactis helleborine	-30.34 ± 1.58	7.38 ± 5.60
Epipactis neglecta	-27.31 ± 1.27	19.04 ± 0.84
Fragaria vesca	-30.80 ± 0.46	-5.16 ± 1.22
Helleborus foetidus	-30.94 ± 0.36	-3.22 ± 1.61
Neottia nidus-avis	-23.10 ± 0.39	7.11 ± 1.23
Plantanthera chlorantha	-30.79 ± 0.66	-3.07 ± 0.97
Rubus fruticosus	-31.63 ± 0.85	-5.01 ± 0.77
Vincetoxicum hirundinaria	-32.41 ± 0.29	-3.75 ± 1.10
Site 2		
Epipactis helleborine	-32.02 ± 0.14	1.16 ± 1.30
Epipactis neglecta	-27.31 ± 0.54	16.81 ± 2.75
Geum urbanum	-32.25 ± 0.43	-7.62 ± 0.24
Lamium galeobdolon	-32.83 ± 0.63	-7.21 ± 0.28
Mercuralis perennis	-31.33 ± 0.50	-2.71 ± 0.46
Neottia nidus-avis	-23.13 ± 0.29	10.80 ± 0.84
Rubus fruticosus	-32.16 ± 0.47	-6.30 ± 0.56
Stachys sylvatica	-33.74 ± 0.65	-4.61 ± 0.55
Vicia sepium	-32.18 ± 0.33	-3.11 ± 0.22
